# P04-12 The efficacy and feasibility of the SPRINTT physical activity intervention in Finland

**DOI:** 10.1093/eurpub/ckac095.066

**Published:** 2022-08-29

**Authors:** Annele Urtamo, Satu Jyväkorpi, Hanna Öhman, Timo Strandberg

**Affiliations:** Department of General Practice and Primary Health Care, University of Helsinki, Finland

**Keywords:** Older adults, frailty, disability, physical exercise

## Abstract

**Background:**

Older adults with frailty and sarcopenia are at high risk of disability. These older adults get benefit from physical activity. However, it is challenging to get them involved in exercise interventions. The SPRINtT trial has investigated the efficacy and feasibility of a multicomponent intervention in the prevention of mobility disability in older adults with frailty and sarcopenia in 11 European countries, under the coordination of the Università Cattolica del Sacro Cuore, Italy. Altogether 1566 candidates were recruited to the SPRINtT RCT, and 142 of them in Finland.

**Methods:**

The participants (n = 70) completed at least two years of physical activity training. The training was performed at moderate intensity and consisted of walking, strength, balance and flexibility exercises. The participants attended training two times a week at the center with the addition of home-based exercises. The training intensity increased gradually. The primary outcome of mobility disability was operationalized as an inability to complete the 400-m walk test. Secondary outcomes of physical performance were the short physical performance battery (SPPB) and handgrip strength.

**Results:**

The results of the intervention will be revealed in spring 2020. Participants experienced that their physical performance improved during the follow-up. The physical activity program and the home-based exercises can be performed without any equipment and could, therefore, be easily implemented for practice.

**Conclusions:**

The physical activity intervention was feasible and could be further recommended for older people with sarcopenia and physical frailty if the final results support these experiences.

